# 3-(2-Amino­ethyl)-2-(4-chloro­anilino)­quinazolin-4(3*H*)-one methanol 0.75-solvate

**DOI:** 10.1107/S160053680804049X

**Published:** 2008-12-07

**Authors:** Xu-Hong Yang, Xiao-Bao Chen, Si-Xuan Zhou

**Affiliations:** a Department of Chemistry and Life Science, Xianning College, Xianning 4371000, Hubei, People’s Republic of China; b Department of Medicinal Chemistry, Yunyang Medical College, Shiyan 442000, Hubei, People’s Republic of China; c School of Chemistry and Chemical Engineering, Hunan University, Changsha 410082, Hunan, People’s Republic of China

## Abstract

In the asymmetric unit of the title compound, C_16_H_15_ClN_4_O·0.75CH_3_OH, there are two independent quinazolin-4(3*H*)-one mol­ecules and one and a half methanol mol­ecules. One of the methanol mol­ecules is disordered over two positions with equal occupancies. The dihedral angles between the quinazoline ring system and the chloro­benzene ring in the two quinazolin-4(3*H*)-one mol­ecules are essentially the same, at 39.83 (1) and 39.84 (1)°. Intra­molecular N—H⋯N and O—H⋯O, and inter­molecular N—H⋯O and N—H⋯N hydrogen bonds are observed. In addition, π–π stacking inter­actions, with centroid-to-centroid distances of 3.654 (1), 3.766 (1) and 3.767 (1) Å, and weak C—H⋯π inter­actions, are observed.

## Related literature

For the biological activity of quinazoline-4(3*H*)-one derivatives, see: Bartroli *et al.* (1998 ▶); Kung *et al.* (1999 ▶); Malamas & Millen (1991 ▶); Mannschreck *et al.* (1984 ▶); Matsuno *et al.* (2002 ▶); Palmer *et al.* (1997 ▶); Pandeya *et al.* (1999 ▶); Shiba *et al.* (1997 ▶); Tsou *et al.* (2001 ▶). For the synthesis of the title compound, see: Hu *et al.* (2006 ▶); Yang *et al.* (2008 ▶).
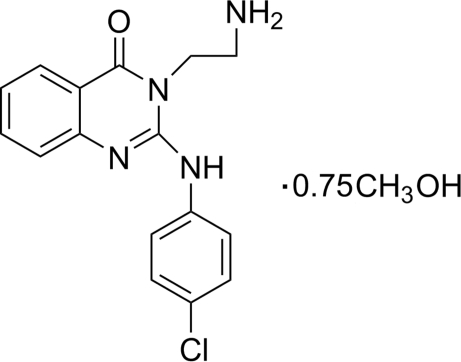


## Experimental

### 

#### Crystal data

C_16_H_15_ClN_4_O·0.75CH_4_O
*M* *_r_* = 338.81
Monoclinic, 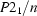

*a* = 13.380 (3) Å
*b* = 12.048 (2) Å
*c* = 21.105 (4) Å
β = 104.49 (3)°
*V* = 3293.8 (11) Å^3^
*Z* = 8
Mo *K*α radiation
μ = 0.25 mm^−1^
*T* = 293 (2) K
0.23 × 0.20 × 0.10 mm

#### Data collection

Bruker SMART APEX CCD area-detector diffractometer
Absorption correction: none
18726 measured reflections
6451 independent reflections
4529 reflections with *I* > 2σ(*I*)
*R*_int_ = 0.018

#### Refinement

*R*[*F* ^2^ > 2σ(*F* ^2^)] = 0.045
*wR*(*F* ^2^) = 0.132
*S* = 1.07
6451 reflections
453 parameters
9 restraints
H atoms treated by a mixture of independent and constrained refinement
Δρ_max_ = 0.22 e Å^−3^
Δρ_min_ = −0.40 e Å^−3^

### 

Data collection: *SMART* (Bruker, 2000 ▶); cell refinement: *SAINT* (Bruker, 2000 ▶); data reduction: *SAINT*; program(s) used to solve structure: *SHELXS97* (Sheldrick, 2008 ▶); program(s) used to refine structure: *SHELXL97* (Sheldrick, 2008 ▶); molecular graphics: *SHELXTL* (Sheldrick, 2008 ▶); software used to prepare material for publication: *SHELXTL*.

## Supplementary Material

Crystal structure: contains datablocks global, I. DOI: 10.1107/S160053680804049X/is2361sup1.cif

Structure factors: contains datablocks I. DOI: 10.1107/S160053680804049X/is2361Isup2.hkl

Additional supplementary materials:  crystallographic information; 3D view; checkCIF report

## Figures and Tables

**Table 1:** Hydrogen-bond geometry (Å, °)

*D*—H⋯*A*	*D*—H	H⋯*A*	*D*⋯*A*	*D*—H⋯*A*
N5—H5*A*⋯N8	0.863 (9)	1.933 (11)	2.787 (3)	170 (2)
O4—H4*C*⋯O1	0.82	2.15	2.889 (6)	151
O3—H3*A*⋯O2	0.835 (10)	1.917 (11)	2.744 (3)	170 (2)
N1—H1*A*⋯N4	0.877 (9)	1.904 (11)	2.760 (3)	164 (2)
N8—H8*B*⋯O4^i^	0.851 (10)	2.467 (14)	3.284 (8)	161 (2)
N8—H8*A*⋯N2^ii^	0.861 (10)	2.440 (17)	3.160 (2)	142 (2)
N4—H4*B*⋯O3^iii^	0.858 (10)	2.097 (11)	2.941 (3)	168 (2)
N4—H4*A*⋯N6^iv^	0.853 (10)	2.491 (17)	3.223 (3)	144 (2)
C5—H5⋯*Cg*4^iv^	0.93	2.81	3.375 (2)	120
C17—H17⋯*Cg*1^ii^	0.93	2.84	3.459 (2)	125
C32—H32*A*⋯*Cg*2^ii^	0.97	2.98	3.824 (3)	146

Symmetry codes: (i) 
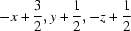
; (ii) 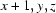; (iii) 
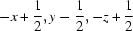
; (iv) 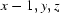. *Cg*1, *Cg*2 and *Cg*4 are the centroids of the N2/C7/N3/C8–C10, C1–C6 and N6/C23/N7/C24–C26 rings, respectively.

**Fig. 1.**
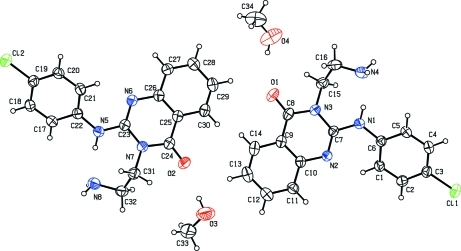
View of the molecular structure of (I), showing the atom labelling scheme and with displacement ellipsoids drawn at the 50% probability level.

**Fig. 2.**
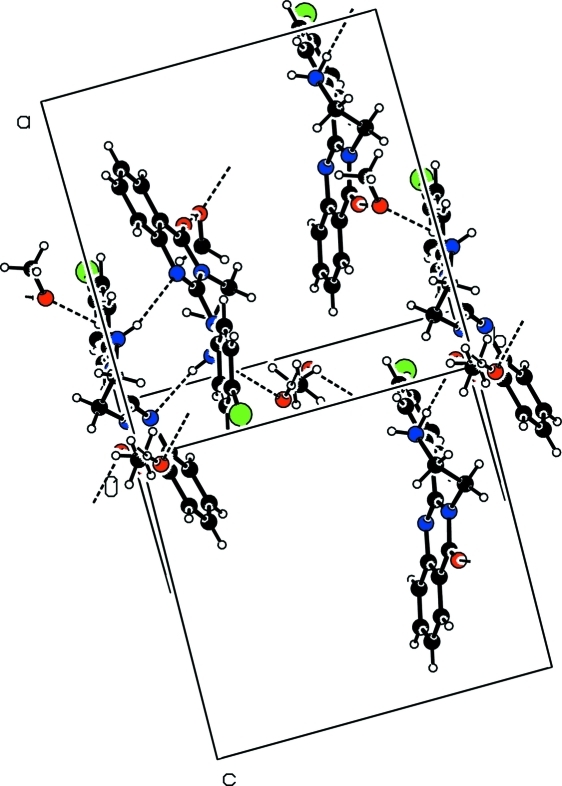
A partial view of the crystal packing of (I), showing the formation of N—H···N, O—H···O and N—H···O hydrogen-bonds (dashed lines).

## supplementary crystallographic information

### Comment

Quinazoline-4(3*H*)-one derivatives have broad biological properties. Some of these activities include antimicrobial (Pandeya *et al.*, 1999; Shiba *et al.*, 1997), antidiabetic (Malamas & Millen, 1991), anticonvulsant (Mannschreck *et al.*, 1984), antibacterial (Kung *et al.*, 1999), antifungal (Bartroli *et al.*, 1998), protein tyrosine kinase inhibitors (Palmer *et al.*, 1997), EGFR inhibitors (Tsou *et al.*, 2001) and PDGFR phosphorylation inhibitors (Matsuno *et al.*, 2002). We have recently focused on the synthesis of heterocyclic compounds using an aza-Wittig reaction. We have reported the synthesis of the title compound (Yang *et al.*, 2008). We present here the crystal structure of the title compound, (I) (Fig. 1), which can be used as a precursor for obtaining bioactive molecules.

In the crystal structure, there are two quinazolin-4(3*H*)-one molecules in the asymmetric unit. The quinazoline heterocycle and the adjacent chlorobenzene ring are not planar, but inclined at 39.83 (1)°. Significant intramolecular N—H···N and O—H···O and intermolecular N—H···O and N—H···N hydrogen bonds contribute strongly to the stability of the molecular configuration (Fig. 2 and Table 1). The crystal structure (Fig. 2) is stabilized by weak intermolecular C—H···π hydrogen bonds (Table 1) and by π–π stacking interactions with centroid–centroid separations of 3.654 (1), 3.767 (1) and 3.766 (1) Å for *Cg*1···*Cg*5^i^, *Cg*2···*Cg*6^ii^ and *Cg*3···*Cg*5^i^, respectively, where *Cg*1, *Cg*2, *Cg*3, *Cg*5 and *Cg*6 are the centroids of rings N2/C7/N3/C8–C10, C1–C6, C9–C14, C17–C22 and C25–C30, respectively [symmetry codes: (i) 1/2 - *x*, -1/2 + *y*, 1/2 - *z*, (ii) 3/2 - *x*, -1/2 + *y*, 1/2 - *z*].

### Experimental

The title compound was prepared according to the literature method (Hu *et al.*,  2006; Yang *et al.*, 2008). Single crystals suitable for X-ray diffraction were obtained from a methanol–dichloromethane (1:1 *v*/*v*) solution at room temperature.

### Refinement

One of the methanol solvent molecules was found to be disordered over two positions. Their final occupancies were set to be 0.5:0.5. H atoms bonded to C were placed at calculated positions, with C—H distances of 0.97 and 0.93 Å for H atoms bonded to *sp*^3^ and *sp*^2^ C atoms, respectively. They were refined using a riding model, with *U*_iso_(H) = 1.2*U*_eq_(C) or 1.5*U*_eq_(methyl C). H atoms bonded to N and O atoms were found in a difference map and then refined with distance restraints of N—H = 0.86 (2) Å and O—H = 0.82 (2) Å, and with *U*_iso_(H) = 1.2*U*_eq_(N) and 1.5*U*_eq_(O).

### Crystal data

**Table:**

C_16_H_15_ClN_4_O·0.75CH_4_O	*F*(000) = 1420
*M**_r_* = 338.81	*D*_x_ = 1.366 Mg m^−^^3^
Monoclinic, *P*2_1_/*n*	Mo *K*α radiation, λ = 0.71073 Å
*a* = 13.380 (3) Å	Cell parameters from 5048 reflections
*b* = 12.048 (2) Å	θ = 2.2–25.8°
*c* = 21.105 (4) Å	µ = 0.25 mm^−^^1^
β = 104.49 (3)°	*T* = 293 K
*V* = 3293.8 (11) Å^3^	Block, colourless
*Z* = 8	0.23 × 0.20 × 0.10 mm

### Data collection

**Table:**

Bruker SMART APEX CCD area-detector diffractometer	4529 reflections with *I* > 2σ(*I*)
Radiation source: fine-focus sealed tube	*R*_int_ = 0.018
graphite	θ_max_ = 26.0°, θ_min_ = 2.0°
φ and ω scans	*h* = −16→16
18726 measured reflections	*k* = −13→14
6451 independent reflections	*l* = −25→23

### Refinement

**Table:**

Refinement on *F*^2^	Primary atom site location: structure-invariant direct methods
Least-squares matrix: full	Secondary atom site location: difference Fourier map
*R*[*F*^2^ > 2σ(*F*^2^)] = 0.045	Hydrogen site location: inferred from neighbouring sites
*wR*(*F*^2^) = 0.132	H atoms treated by a mixture of independent and constrained refinement
*S* = 1.07	*w* = 1/[σ^2^(*F*_o_^2^) + (0.0651*P*)^2^ + 0.4596*P*] where *P* = (*F*_o_^2^ + 2*F*_c_^2^)/3
6451 reflections	(Δ/σ)_max_ = 0.001
453 parameters	Δρ_max_ = 0.22 e Å^−^^3^
9 restraints	Δρ_min_ = −0.40 e Å^−^^3^

### Special details

**Table:**

Geometry. All e.s.d.'s (except the e.s.d. in the dihedral angle between two l.s. planes) are estimated using the full covariance matrix. The cell e.s.d.'s are taken into account individually in the estimation of e.s.d.'s in distances, angles and torsion angles; correlations between e.s.d.'s in cell parameters are only used when they are defined by crystal symmetry. An approximate (isotropic) treatment of cell e.s.d.'s is used for estimating e.s.d.'s involving l.s. planes.
Refinement. Refinement of *F*^2^ against ALL reflections. The weighted *R*-factor *wR* and goodness of fit *S* are based on *F*^2^, conventional *R*-factors *R* are based on *F*, with *F* set to zero for negative *F*^2^. The threshold expression of *F*^2^ > σ(*F*^2^) is used only for calculating *R*-factors(gt) *etc*. and is not relevant to the choice of reflections for refinement. *R*-factors based on *F*^2^ are statistically about twice as large as those based on *F*, and *R*- factors based on ALL data will be even larger.

### Fractional atomic coordinates and isotropic or equivalent isotropic displacement parameters (Å^2^)

**Table:**

	*x*	*y*	*z*	*U*_iso_*/*U*_eq_	Occ. (<1)
C1	−0.08984 (15)	0.47979 (16)	0.12395 (9)	0.0528 (5)	
H1	−0.0347	0.4622	0.1063	0.063*	
C2	−0.18874 (15)	0.48069 (16)	0.08437 (10)	0.0556 (5)	
H2	−0.2003	0.4636	0.0402	0.067*	
C3	−0.27035 (14)	0.50686 (16)	0.11023 (10)	0.0534 (5)	
C4	−0.25485 (14)	0.53302 (15)	0.17510 (10)	0.0530 (5)	
H4	−0.3104	0.5514	0.1921	0.064*	
C5	−0.15634 (14)	0.53178 (14)	0.21477 (9)	0.0489 (4)	
H5	−0.1456	0.5491	0.2589	0.059*	
C6	−0.07211 (14)	0.50494 (14)	0.18997 (9)	0.0458 (4)	
C7	0.11714 (13)	0.52077 (14)	0.22075 (9)	0.0462 (4)	
C8	0.30176 (14)	0.52546 (15)	0.26820 (10)	0.0534 (5)	
C9	0.30837 (13)	0.57442 (15)	0.20688 (10)	0.0499 (4)	
C10	0.21855 (13)	0.59011 (14)	0.15719 (9)	0.0481 (4)	
C11	0.22667 (16)	0.63532 (17)	0.09793 (10)	0.0593 (5)	
H11	0.1675	0.6467	0.0644	0.071*	
C12	0.32126 (18)	0.66313 (18)	0.08869 (12)	0.0688 (6)	
H12	0.3256	0.6935	0.0490	0.083*	
C13	0.41067 (18)	0.64669 (19)	0.13779 (13)	0.0729 (6)	
H13	0.4745	0.6655	0.1309	0.087*	
C14	0.40457 (15)	0.60298 (17)	0.19601 (12)	0.0642 (6)	
H14	0.4645	0.5918	0.2289	0.077*	
C15	0.19411 (16)	0.42811 (17)	0.32874 (10)	0.0621 (5)	
H15A	0.2586	0.3890	0.3457	0.074*	
H15B	0.1405	0.3729	0.3142	0.074*	
C16	0.1693 (2)	0.4950 (2)	0.38362 (11)	0.0721 (6)	
H16A	0.1837	0.4505	0.4232	0.087*	
H16B	0.2133	0.5600	0.3920	0.087*	
C17	1.20193 (15)	0.78133 (15)	0.30649 (10)	0.0524 (5)	
H17	1.1943	0.7627	0.2628	0.063*	
C18	1.29879 (15)	0.78222 (16)	0.34860 (10)	0.0560 (5)	
H18	1.3565	0.7649	0.3335	0.067*	
C19	1.30922 (15)	0.80899 (17)	0.41320 (11)	0.0572 (5)	
C20	1.22461 (15)	0.83549 (17)	0.43566 (10)	0.0599 (5)	
H20	1.2327	0.8534	0.4795	0.072*	
C21	1.12771 (15)	0.83567 (16)	0.39363 (10)	0.0572 (5)	
H21	1.0706	0.8544	0.4091	0.069*	
C22	1.11498 (14)	0.80811 (14)	0.32859 (9)	0.0478 (4)	
C23	0.92555 (14)	0.79103 (14)	0.29335 (9)	0.0483 (4)	
C24	0.74195 (14)	0.78926 (15)	0.24330 (10)	0.0517 (5)	
C25	0.73119 (14)	0.74191 (15)	0.30396 (9)	0.0486 (4)	
C26	0.81934 (14)	0.72442 (15)	0.35473 (9)	0.0500 (4)	
C27	0.80719 (17)	0.67926 (18)	0.41340 (11)	0.0629 (5)	
H27	0.8649	0.6665	0.4477	0.075*	
C28	0.71130 (18)	0.65384 (19)	0.42065 (12)	0.0708 (6)	
H28	0.7045	0.6235	0.4599	0.085*	
C29	0.62350 (16)	0.67269 (18)	0.37022 (12)	0.0667 (6)	
H29	0.5585	0.6554	0.3758	0.080*	
C30	0.63343 (15)	0.71662 (16)	0.31264 (11)	0.0582 (5)	
H30	0.5749	0.7299	0.2789	0.070*	
C31	0.85432 (16)	0.88363 (16)	0.18470 (10)	0.0604 (5)	
H31A	0.9088	0.9377	0.1997	0.072*	
H31B	0.7909	0.9242	0.1668	0.072*	
C32	0.87963 (17)	0.81504 (19)	0.13073 (10)	0.0657 (6)	
H32A	0.8374	0.7485	0.1238	0.079*	
H32B	0.8637	0.8574	0.0903	0.079*	
C33	0.5966 (3)	0.8449 (4)	0.02991 (16)	0.1534 (17)	
H33A	0.6527	0.8967	0.0355	0.230*	
H33B	0.5503	0.8552	−0.0124	0.230*	
H33C	0.6232	0.7706	0.0335	0.230*	
Cl1	−0.39482 (4)	0.50595 (6)	0.05930 (3)	0.0837 (2)	
Cl2	1.43110 (4)	0.81046 (7)	0.46778 (3)	0.0904 (2)	
N1	0.02479 (12)	0.49709 (14)	0.23440 (8)	0.0541 (4)	
H1A	0.0250 (17)	0.5023 (17)	0.2759 (5)	0.065*	
N2	0.12216 (11)	0.56474 (13)	0.16588 (7)	0.0486 (4)	
N3	0.20303 (11)	0.49523 (12)	0.27173 (7)	0.0486 (4)	
H3A	0.5847 (15)	0.8370 (17)	0.1097 (7)	0.215*	
N4	0.06204 (17)	0.52954 (17)	0.36770 (9)	0.0716 (5)	
H4A	0.0474 (18)	0.5956 (11)	0.3769 (12)	0.086*	
H4B	0.0261 (17)	0.4888 (18)	0.3870 (11)	0.086*	
N5	1.01963 (12)	0.81229 (14)	0.28200 (8)	0.0553 (4)	
H5A	1.0179 (16)	0.8063 (17)	0.2410 (5)	0.066*	
N6	0.91717 (11)	0.74695 (13)	0.34781 (8)	0.0524 (4)	
N7	0.84205 (12)	0.81837 (12)	0.24149 (7)	0.0490 (4)	
N8	0.98838 (15)	0.78405 (16)	0.14741 (9)	0.0662 (5)	
H8A	1.0023 (18)	0.7195 (11)	0.1344 (11)	0.079*	
H8B	1.0213 (16)	0.8306 (15)	0.1300 (11)	0.079*	
O1	0.37637 (11)	0.50823 (12)	0.31482 (8)	0.0725 (4)	
O2	0.66919 (11)	0.80647 (12)	0.19558 (7)	0.0678 (4)	
O3	0.5479 (2)	0.8615 (2)	0.07449 (11)	0.1432 (10)	
C34	0.5063 (2)	0.4916 (2)	0.50408 (11)	0.131 (2)	0.50
H34A	0.5228	0.5678	0.4981	0.197*	0.50
H34B	0.5664	0.4465	0.5069	0.197*	0.50
H34C	0.4840	0.4844	0.5437	0.197*	0.50
O4	0.4296 (5)	0.4506 (6)	0.4520 (3)	0.168 (2)	0.50
H4C	0.4324	0.4827	0.4183	0.253*	0.50

### Atomic displacement parameters (Å^2^)

**Table:**

	*U*^11^	*U*^22^	*U*^33^	*U*^12^	*U*^13^	*U*^23^
C1	0.0460 (10)	0.0627 (12)	0.0515 (12)	0.0002 (8)	0.0153 (9)	−0.0065 (9)
C2	0.0538 (11)	0.0646 (12)	0.0466 (11)	−0.0023 (9)	0.0094 (9)	−0.0065 (9)
C3	0.0412 (10)	0.0549 (11)	0.0620 (13)	−0.0048 (8)	0.0086 (9)	0.0037 (9)
C4	0.0465 (10)	0.0491 (10)	0.0682 (13)	−0.0022 (8)	0.0232 (9)	−0.0019 (9)
C5	0.0523 (11)	0.0473 (10)	0.0508 (11)	−0.0065 (8)	0.0197 (9)	−0.0058 (8)
C6	0.0443 (10)	0.0450 (10)	0.0478 (11)	−0.0036 (7)	0.0111 (8)	−0.0012 (8)
C7	0.0424 (10)	0.0478 (10)	0.0461 (11)	0.0011 (7)	0.0066 (8)	−0.0042 (8)
C8	0.0464 (11)	0.0457 (10)	0.0627 (13)	0.0047 (8)	0.0039 (9)	−0.0043 (9)
C9	0.0450 (10)	0.0423 (10)	0.0615 (12)	0.0009 (8)	0.0115 (9)	−0.0078 (8)
C10	0.0462 (10)	0.0442 (10)	0.0550 (11)	0.0008 (8)	0.0149 (8)	−0.0072 (8)
C11	0.0618 (12)	0.0634 (12)	0.0552 (12)	−0.0011 (10)	0.0191 (10)	−0.0021 (10)
C12	0.0767 (15)	0.0650 (13)	0.0743 (15)	−0.0069 (11)	0.0368 (13)	−0.0032 (11)
C13	0.0625 (14)	0.0639 (13)	0.103 (2)	−0.0068 (11)	0.0408 (14)	−0.0064 (13)
C14	0.0447 (11)	0.0554 (12)	0.0912 (17)	−0.0010 (9)	0.0145 (11)	−0.0084 (11)
C15	0.0648 (13)	0.0555 (12)	0.0608 (13)	0.0046 (9)	0.0061 (10)	0.0142 (10)
C16	0.0844 (16)	0.0790 (15)	0.0473 (12)	−0.0093 (12)	0.0059 (11)	0.0110 (11)
C17	0.0589 (12)	0.0484 (10)	0.0532 (11)	−0.0026 (8)	0.0200 (9)	−0.0015 (8)
C18	0.0488 (11)	0.0569 (12)	0.0660 (13)	0.0018 (8)	0.0214 (10)	0.0047 (10)
C19	0.0486 (11)	0.0600 (12)	0.0617 (13)	−0.0071 (9)	0.0111 (9)	0.0072 (10)
C20	0.0562 (12)	0.0727 (13)	0.0518 (12)	−0.0147 (10)	0.0156 (10)	−0.0056 (10)
C21	0.0522 (11)	0.0644 (12)	0.0588 (13)	−0.0074 (9)	0.0208 (10)	−0.0108 (10)
C22	0.0484 (10)	0.0424 (10)	0.0531 (11)	−0.0058 (8)	0.0139 (9)	0.0006 (8)
C23	0.0477 (10)	0.0442 (10)	0.0516 (11)	−0.0012 (8)	0.0097 (9)	−0.0036 (8)
C24	0.0493 (11)	0.0482 (10)	0.0549 (12)	0.0033 (8)	0.0078 (9)	−0.0086 (9)
C25	0.0479 (10)	0.0437 (10)	0.0534 (11)	−0.0010 (8)	0.0114 (9)	−0.0076 (8)
C26	0.0483 (10)	0.0466 (10)	0.0550 (11)	−0.0017 (8)	0.0128 (9)	−0.0042 (8)
C27	0.0569 (12)	0.0735 (14)	0.0566 (13)	−0.0041 (10)	0.0113 (10)	0.0064 (10)
C28	0.0678 (14)	0.0782 (15)	0.0711 (15)	−0.0055 (11)	0.0261 (12)	0.0073 (12)
C29	0.0541 (12)	0.0679 (14)	0.0820 (16)	−0.0083 (10)	0.0242 (12)	−0.0017 (11)
C30	0.0473 (11)	0.0568 (12)	0.0678 (14)	−0.0025 (9)	0.0092 (10)	−0.0080 (10)
C31	0.0619 (12)	0.0540 (12)	0.0634 (13)	0.0021 (9)	0.0124 (10)	0.0117 (10)
C32	0.0673 (14)	0.0764 (14)	0.0520 (12)	−0.0076 (11)	0.0124 (10)	0.0101 (10)
C33	0.107 (3)	0.280 (6)	0.075 (2)	0.029 (3)	0.025 (2)	0.000 (3)
Cl1	0.0437 (3)	0.1160 (5)	0.0841 (4)	−0.0049 (3)	0.0024 (3)	0.0082 (3)
Cl2	0.0523 (3)	0.1351 (6)	0.0773 (4)	−0.0083 (3)	0.0042 (3)	0.0178 (4)
N1	0.0467 (9)	0.0715 (11)	0.0437 (9)	−0.0048 (7)	0.0104 (8)	−0.0004 (8)
N2	0.0438 (8)	0.0563 (9)	0.0450 (9)	0.0024 (7)	0.0098 (7)	−0.0009 (7)
N3	0.0474 (9)	0.0474 (8)	0.0481 (9)	0.0028 (6)	0.0066 (7)	0.0024 (7)
N4	0.0872 (14)	0.0732 (13)	0.0557 (11)	0.0059 (11)	0.0204 (10)	0.0053 (10)
N5	0.0497 (9)	0.0676 (10)	0.0486 (9)	−0.0061 (7)	0.0122 (8)	−0.0009 (8)
N6	0.0461 (9)	0.0573 (9)	0.0524 (10)	−0.0024 (7)	0.0095 (7)	0.0008 (8)
N7	0.0504 (9)	0.0454 (8)	0.0503 (9)	0.0015 (7)	0.0112 (7)	0.0008 (7)
N8	0.0703 (12)	0.0699 (12)	0.0611 (12)	0.0019 (10)	0.0214 (9)	0.0070 (9)
O1	0.0518 (8)	0.0808 (10)	0.0731 (10)	0.0075 (7)	−0.0065 (8)	0.0072 (8)
O2	0.0562 (8)	0.0828 (10)	0.0571 (9)	0.0043 (7)	0.0004 (7)	0.0008 (7)
O3	0.153 (2)	0.196 (3)	0.0823 (15)	0.088 (2)	0.0324 (15)	0.0356 (16)
C34	0.187 (7)	0.107 (4)	0.098 (4)	−0.014 (4)	0.033 (4)	0.004 (3)
O4	0.170 (6)	0.180 (6)	0.120 (4)	0.016 (4)	−0.028 (4)	0.018 (4)

### Geometric parameters (Å, °)

**Table:**

C1—C2	1.377 (3)	C20—H20	0.9300
C1—C6	1.387 (3)	C21—C22	1.381 (3)
C1—H1	0.9300	C21—H21	0.9300
C2—C3	1.374 (3)	C22—N5	1.403 (2)
C2—H2	0.9300	C23—N6	1.296 (2)
C3—C4	1.369 (3)	C23—N5	1.363 (2)
C3—Cl1	1.742 (2)	C23—N7	1.394 (2)
C4—C5	1.373 (3)	C24—O2	1.231 (2)
C4—H4	0.9300	C24—N7	1.394 (2)
C5—C6	1.394 (3)	C24—C25	1.441 (3)
C5—H5	0.9300	C25—C26	1.397 (3)
C6—N1	1.400 (2)	C25—C30	1.399 (3)
C7—N2	1.290 (2)	C26—N6	1.380 (2)
C7—N1	1.367 (2)	C26—C27	1.399 (3)
C7—N3	1.398 (2)	C27—C28	1.364 (3)
C8—O1	1.231 (2)	C27—H27	0.9300
C8—N3	1.390 (2)	C28—C29	1.392 (3)
C8—C9	1.445 (3)	C28—H28	0.9300
C9—C10	1.396 (3)	C29—C30	1.362 (3)
C9—C14	1.406 (3)	C29—H29	0.9300
C10—N2	1.381 (2)	C30—H30	0.9300
C10—C11	1.393 (3)	C31—N7	1.477 (2)
C11—C12	1.369 (3)	C31—C32	1.513 (3)
C11—H11	0.9300	C31—H31A	0.9700
C12—C13	1.387 (3)	C31—H31B	0.9700
C12—H12	0.9300	C32—N8	1.457 (3)
C13—C14	1.358 (3)	C32—H32A	0.9700
C13—H13	0.9300	C32—H32B	0.9700
C14—H14	0.9300	C33—O3	1.287 (4)
C15—N3	1.479 (2)	C33—H33A	0.9600
C15—C16	1.514 (3)	C33—H33B	0.9600
C15—H15A	0.9700	C33—H33C	0.9600
C15—H15B	0.9700	N1—H1A	0.877 (9)
C16—N4	1.451 (3)	N4—H4A	0.853 (10)
C16—H16A	0.9700	N4—H4B	0.858 (10)
C16—H16B	0.9700	N5—H5A	0.863 (9)
C17—C18	1.376 (3)	N8—H8A	0.861 (10)
C17—C22	1.395 (3)	N8—H8B	0.851 (10)
C17—H17	0.9300	O3—H3A	0.835 (10)
C18—C19	1.374 (3)	C34—O4	1.392 (6)
C18—H18	0.9300	C34—H34A	0.9600
C19—C20	1.370 (3)	C34—H34B	0.9600
C19—Cl2	1.745 (2)	C34—H34C	0.9600
C20—C21	1.376 (3)	O4—H4C	0.8200
			
C2—C1—C6	120.30 (18)	N6—C23—N5	121.41 (17)
C2—C1—H1	119.8	N6—C23—N7	124.26 (17)
C6—C1—H1	119.8	N5—C23—N7	114.31 (17)
C3—C2—C1	119.97 (18)	O2—C24—N7	120.26 (19)
C3—C2—H2	120.0	O2—C24—C25	124.01 (18)
C1—C2—H2	120.0	N7—C24—C25	115.72 (17)
C4—C3—C2	120.89 (18)	C26—C25—C30	120.28 (18)
C4—C3—Cl1	120.05 (15)	C26—C25—C24	119.23 (17)
C2—C3—Cl1	119.06 (16)	C30—C25—C24	120.47 (18)
C3—C4—C5	119.29 (17)	N6—C26—C25	122.04 (18)
C3—C4—H4	120.4	N6—C26—C27	119.58 (18)
C5—C4—H4	120.4	C25—C26—C27	118.36 (17)
C4—C5—C6	121.07 (17)	C28—C27—C26	120.5 (2)
C4—C5—H5	119.5	C28—C27—H27	119.8
C6—C5—H5	119.5	C26—C27—H27	119.8
C1—C6—C5	118.47 (17)	C27—C28—C29	121.0 (2)
C1—C6—N1	123.70 (17)	C27—C28—H28	119.5
C5—C6—N1	117.66 (16)	C29—C28—H28	119.5
N2—C7—N1	121.83 (16)	C30—C29—C28	119.57 (19)
N2—C7—N3	124.32 (16)	C30—C29—H29	120.2
N1—C7—N3	113.83 (16)	C28—C29—H29	120.2
O1—C8—N3	120.28 (19)	C29—C30—C25	120.30 (19)
O1—C8—C9	124.43 (19)	C29—C30—C14	101.64 (14)
N3—C8—C9	115.28 (17)	C25—C30—C14	129.96 (14)
C10—C9—C14	119.71 (19)	C29—C30—H30	119.8
C10—C9—C8	119.60 (16)	C25—C30—H30	119.8
C14—C9—C8	120.66 (18)	N7—C31—C32	114.31 (16)
N2—C10—C11	119.40 (17)	N7—C31—H31A	108.7
N2—C10—C9	121.80 (17)	C32—C31—H31A	108.7
C11—C10—C9	118.78 (17)	N7—C31—H31B	108.7
C12—C11—C10	120.4 (2)	C32—C31—H31B	108.7
C12—C11—H11	119.8	H31A—C31—H31B	107.6
C10—C11—H11	119.8	N8—C32—C31	110.86 (18)
C11—C12—C13	120.9 (2)	N8—C32—H32A	109.5
C11—C12—H12	119.5	C31—C32—H32A	109.5
C13—C12—H12	119.5	N8—C32—H32B	109.5
C14—C13—C12	119.7 (2)	C31—C32—H32B	109.5
C14—C13—H13	120.1	H32A—C32—H32B	108.1
C12—C13—H13	120.1	O3—C33—H33A	109.5
C13—C14—C9	120.5 (2)	O3—C33—H33B	109.5
C13—C14—C30	103.46 (15)	H33A—C33—H33B	109.5
C9—C14—C30	128.66 (15)	O3—C33—H33C	109.5
C13—C14—H14	119.8	H33A—C33—H33C	109.5
C9—C14—H14	119.8	H33B—C33—H33C	109.5
N3—C15—C16	114.16 (17)	C7—N1—C6	125.38 (16)
N3—C15—H15A	108.7	C7—N1—H1A	114.7 (15)
C16—C15—H15A	108.7	C6—N1—H1A	115.8 (15)
N3—C15—H15B	108.7	C7—N2—C10	118.03 (16)
C16—C15—H15B	108.7	C8—N3—C7	120.67 (16)
H15A—C15—H15B	107.6	C8—N3—C15	117.26 (16)
N4—C16—C15	111.68 (19)	C7—N3—C15	121.90 (15)
N4—C16—H16A	109.3	C16—N4—H4A	119.0 (17)
C15—C16—H16A	109.3	C16—N4—H4B	111.4 (17)
N4—C16—H16B	109.3	H4A—N4—H4B	104 (2)
C15—C16—H16B	109.3	C23—N5—C22	126.00 (17)
H16A—C16—H16B	107.9	C23—N5—H5A	111.5 (15)
C18—C17—C22	120.78 (18)	C22—N5—H5A	119.4 (15)
C18—C17—H17	119.6	C23—N6—C26	117.98 (16)
C22—C17—H17	119.6	C23—N7—C24	120.39 (16)
C19—C18—C17	119.23 (18)	C23—N7—C31	122.22 (15)
C19—C18—H18	120.4	C24—N7—C31	117.31 (16)
C17—C18—H18	120.4	C32—N8—H8A	115.9 (17)
C20—C19—C18	120.71 (19)	C32—N8—H8B	108.7 (16)
C20—C19—Cl2	119.06 (17)	H8A—N8—H8B	106 (2)
C18—C19—Cl2	120.23 (16)	C24—O2—O3	164.88 (15)
C19—C20—C21	120.29 (19)	C33—O3—O2	110.7 (2)
C19—C20—H20	119.9	C33—O3—H3A	107.5 (17)
C21—C20—H20	119.9	O4—C34—H34A	112.5
C20—C21—C22	120.17 (18)	O4—C34—H34B	106.7
C20—C21—H21	119.9	H34A—C34—H34B	109.5
C22—C21—H21	119.9	O4—C34—H34C	109.1
C21—C22—C17	118.83 (18)	H34A—C34—H34C	109.5
C21—C22—N5	123.52 (17)	H34B—C34—H34C	109.5
C17—C22—N5	117.52 (17)		
			
C6—C1—C2—C3	0.2 (3)	C28—C29—C30—C25	0.5 (3)
C1—C2—C3—C4	0.5 (3)	C28—C29—C30—C14	152.10 (18)
C1—C2—C3—Cl1	−179.32 (15)	C26—C25—C30—C29	−1.3 (3)
C2—C3—C4—C5	−0.7 (3)	C24—C25—C30—C29	−179.48 (18)
Cl1—C3—C4—C5	179.06 (14)	C26—C25—C30—C14	−143.90 (15)
C3—C4—C5—C6	0.3 (3)	C24—C25—C30—C14	37.9 (2)
C2—C1—C6—C5	−0.5 (3)	C13—C14—C30—C29	178.95 (18)
C2—C1—C6—N1	174.61 (17)	C9—C14—C30—C29	29.7 (2)
C4—C5—C6—C1	0.3 (3)	C13—C14—C30—C25	−33.4 (2)
C4—C5—C6—N1	−175.16 (16)	C9—C14—C30—C25	177.3 (2)
O1—C8—C9—C10	179.95 (17)	N7—C31—C32—N8	−78.3 (2)
N3—C8—C9—C10	1.3 (2)	N2—C7—N1—C6	7.5 (3)
O1—C8—C9—C14	2.1 (3)	N3—C7—N1—C6	−173.77 (16)
N3—C8—C9—C14	−176.57 (16)	C1—C6—N1—C7	35.7 (3)
C14—C9—C10—N2	−179.09 (16)	C5—C6—N1—C7	−149.14 (18)
C8—C9—C10—N2	3.0 (3)	N1—C7—N2—C10	177.40 (16)
C14—C9—C10—C11	−0.8 (3)	N3—C7—N2—C10	−1.2 (3)
C8—C9—C10—C11	−178.69 (17)	C11—C10—N2—C7	178.56 (17)
N2—C10—C11—C12	178.72 (17)	C9—C10—N2—C7	−3.2 (3)
C9—C10—C11—C12	0.4 (3)	O1—C8—N3—C7	175.92 (16)
C10—C11—C12—C13	0.2 (3)	C9—C8—N3—C7	−5.4 (2)
C11—C12—C13—C14	−0.3 (3)	O1—C8—N3—C15	−8.7 (3)
C12—C13—C14—C9	−0.1 (3)	C9—C8—N3—C15	170.03 (16)
C12—C13—C14—C30	−152.49 (18)	N2—C7—N3—C8	5.7 (3)
C10—C9—C14—C13	0.7 (3)	N1—C7—N3—C8	−172.99 (15)
C8—C9—C14—C13	178.53 (18)	N2—C7—N3—C15	−169.51 (18)
C10—C9—C14—C30	145.43 (15)	N1—C7—N3—C15	11.8 (2)
C8—C9—C14—C30	−36.7 (2)	C16—C15—N3—C8	97.6 (2)
N3—C15—C16—N4	75.0 (2)	C16—C15—N3—C7	−87.0 (2)
C22—C17—C18—C19	0.5 (3)	N6—C23—N5—C22	−11.2 (3)
C17—C18—C19—C20	−0.5 (3)	N7—C23—N5—C22	170.10 (16)
C17—C18—C19—Cl2	179.84 (14)	C21—C22—N5—C23	−33.2 (3)
C18—C19—C20—C21	0.0 (3)	C17—C22—N5—C23	151.00 (17)
Cl2—C19—C20—C21	179.59 (15)	N5—C23—N6—C26	−177.79 (16)
C19—C20—C21—C22	0.6 (3)	N7—C23—N6—C26	0.8 (3)
C20—C21—C22—C17	−0.6 (3)	C25—C26—N6—C23	3.6 (3)
C20—C21—C22—N5	−176.36 (18)	C27—C26—N6—C23	−178.51 (18)
C18—C17—C22—C21	0.0 (3)	N6—C23—N7—C24	−6.1 (3)
C18—C17—C22—N5	176.04 (16)	N5—C23—N7—C24	172.51 (15)
O2—C24—C25—C26	179.05 (18)	N6—C23—N7—C31	170.51 (17)
N7—C24—C25—C26	−2.5 (2)	N5—C23—N7—C31	−10.8 (2)
O2—C24—C25—C30	−2.7 (3)	O2—C24—N7—C23	−174.86 (16)
N7—C24—C25—C30	175.71 (16)	C25—C24—N7—C23	6.6 (2)
C30—C25—C26—N6	179.15 (16)	O2—C24—N7—C31	8.3 (3)
C24—C25—C26—N6	−2.6 (3)	C25—C24—N7—C31	−170.18 (15)
C30—C25—C26—C27	1.3 (3)	C32—C31—N7—C23	86.7 (2)
C24—C25—C26—C27	179.46 (17)	C32—C31—N7—C24	−96.5 (2)
N6—C26—C27—C28	−178.38 (19)	N7—C24—O2—O3	5.4 (6)
C25—C26—C27—C28	−0.4 (3)	C25—C24—O2—O3	−176.2 (4)
C26—C27—C28—C29	−0.4 (3)	C24—O2—O3—C33	30.1 (7)
C27—C28—C29—C30	0.4 (3)		

### Hydrogen-bond geometry (Å, °)

**Table:**

*D*—H···*A*	*D*—H	H···*A*	*D*···*A*	*D*—H···*A*
N5—H5A···N8	0.86 (1)	1.93 (1)	2.787 (3)	170 (2)
O4—H4C···O1	0.82	2.15	2.889 (6)	151
O3—H3A···O2	0.84 (1)	1.92 (1)	2.744 (3)	170 (2)
N1—H1A···N4	0.88 (1)	1.90 (1)	2.760 (3)	164 (2)
N8—H8B···O4^i^	0.85 (1)	2.47 (1)	3.284 (8)	161 (2)
N8—H8A···N2^ii^	0.86 (1)	2.44 (2)	3.160 (2)	142 (2)
N4—H4B···O3^iii^	0.86 (1)	2.10 (1)	2.941 (3)	168 (2)
N4—H4A···N6^iv^	0.85 (1)	2.49 (2)	3.223 (3)	144 (2)
C5—H5···Cg4^iv^	0.93	2.81	3.375 (2)	120
C17—H17···Cg1^ii^	0.93	2.84	3.459 (2)	125
C32—H32A···Cg2^ii^	0.97	2.98	3.824 (3)	146

Symmetry codes: (i) −*x*+3/2, *y*+1/2, −*z*+1/2; (ii) *x*+1, *y*, *z*; (iii) −*x*+1/2, *y*−1/2, −*z*+1/2; (iv) *x*−1, *y*, *z*.
